# Why should I consult? The impact of social support on patient consultation in online healthcare communities

**DOI:** 10.3389/fpsyg.2022.993088

**Published:** 2022-09-20

**Authors:** Xiaochen Liu, Zhen Xu, Xintao Yu, Tetsuaki Oda

**Affiliations:** ^1^Graduate School of Technology Management, Ritsumeikan University, Osaka, Japan; ^2^School of Communication, East China University of Political Science and Law, Shanghai, China; ^3^School of Economics and Management, Liaoning University of Technology, Jinzhou, China

**Keywords:** online healthcare communities, social support theory, informational support, emotional support, patients’ consultations, patients’ compliments

## Abstract

The COVID-19 epidemic put the traditional healthcare system and offline consultation method under strain. Patient consultations through online healthcare communities (OHCs) provide patients and physicians with a more convenient and secure route. Based on the social support theory, this study explores the impact of three dimensions of social support from physicians—information diagnosticity, source credibility, and emotional support—on patient consultations in OHCs and their moderating effect on patients’ compliments. We utilized Python Spiders to retrieve data from Haodf.com and gathered 2,982 physician reports. The model uses OLS regression with fixed effect estimations. The results show that these three dimensions of social support are positively impacted by consultation. Furthermore, patients’ compliments weaken the positive relationship between the three dimensions of physicians’ social support and patient consultations. This study contributes to the literature on social support theory in OHCs by exploring the physicians’ social support dimension and its impact on patient consultation. Moreover, this study makes practical contributions to physicians and platform administrators in OHCs.

## Introduction

According to previous research in the context of China’s OHCs, experienced physicians and their time and effort are valuable medical resources that are difficult to obtain (Lu et al., 2021). In addition, the global outbreak of COVID-19 has hindered people’s mobility. As a result, telemedicine has thrived in this environment. Online Healthcare Communities (OHCs) are one of the most prevalent telemedicine options. OHCs mix various telemedicine functions and offer a novel method of reaching consensus between physicians and patients. In contrast to traditional medical services, OHCs allow patients to evaluate an abundance of information on multiple physicians and help them further analyze the collected data to choose the physician they prefer to consult (Lu et al., 2021). OHC makes it easy and secure for both physicians and patients to engage, allowing patients to receive health services, social support, and physician information (Chen et al., 2021). However, patients may have a lower level of trust in physicians when they consult with them online rather than in person (Liu et al., 2022). Consequently, academics have focused on discovering which characteristics influence online patient consultations.

In OHCs context, social support plays a critical role for patients and physicians. For patients, social support can improve health outcomes, including health behaviors, physical health, and psychological health (Uchino, 2009). For physicians, social support is also important, because social support significantly affects their online reputations (Wang and Liu, 2022). This is because patients generally gather available information to choose their social support (Jadad et al., 2000). Thus, it is worth investigating the social support provided by physicians as service providers on OHCs platforms. However, there is still a lack of research on the impact of social support on patient consultation from physician’s perspectives. In addition, according to the theory of social support, the two primary kinds of social support include informational and emotional support (Huang et al., 2010). Emotional support can decrease patient’s anxiety and stress (Walther and Boyd, 2002). In the context of OHCs, patients face uncertainty while choosing doctors to consult due to information asymmetry (Wu and Lu, 2016). OHCs offer a mechanism for sharing knowledge and emotional support (Ba and Wang, 2013) without chronological, geographical, or spatial limits (Fan et al., 2014). Online knowledge and assistance offered *via* OHCs boost users’ functional and psychological well-being (Chen et al., 2019). However, there is still a lack of research on the dimensions of physicians’ social support in OHCs. Therefore, it is necessary to explore the dimensions of the physicians’ social support in OHCs and its impact on patient consultation. There is much more physician information in OHCs compared with the traditional consultation environment, making it more difficult for patients to make decisions (Peng et al., 2021). When a physician’s webpage has an excess of information, the information is diluted and the patient’s attention is likely to fail (Ouyang et al., 2022). When patients choose a physician for OHCs consultation, digesting information is time-consuming and requires a lot of effort (Narayanan and Georgiou, 2013). In general, a physician’s reputation, particularly positive patient compliments, enables other patients evaluate the physician’s competence-related information more efficiently (Lu and Wu, 2016). The compliments of other patients in OHCs have shown to be easily recognized and understood by other patients (Wu et al., 2020). However, it is unclear whether the process of choosing physicians in OHCs will be affected by other patients’ compliments. Therefore, it is necessary to explore the role of other patients’ compliments on physicians’ social support information and patients’ decision-making.

In summary, it is found that OHCs have the above research gaps related to social support, patients’ compliments, and patient consultations. To fill these gaps, this study explores the dimensions of physicians’ social support in OHCs and their impact on patient consultations. We obtained data from 2,982 physician from the Haodf.com website using Python and estimated the concept model by OLS regression with fixed effect. This study explores the following three issues: (1) the dimensions of physicians’ social support (informational diagnosticity, source credibility, and emotional support) in OHCs; (2) the impact of those three dimensions of physicians’ social support on patient consultations; and (3) the moderating effect of patients’ compliments. Our research enriches the social support theory in OHCs and helps physicians attract more consultations by optimizing their online information.

## Literature review

### Online healthcare consultation

Increasingly, individuals are turning to the Internet to fulfill health-related requirements due to the rise of Health 2.0 technologies (Chen et al., 2021). Telemedicine is an emerging health communication technology that allows healthcare practitioners to connect with patients *via* audio- and video-capable devices such as computers (Swan et al., 2019). Online healthcare communities (OHCs) have increased due to the development of telemedicine technology. OHCs ensure that healthcare consultations about various diseases and treatments are always available and easy to use. Meanwhile, OHCs members can offer support and knowledge in coping with disease experiences (Yan and Tan, 2014; Mirzaei and Esmaeilzadeh, 2021). In online healthcare consultations, physicians and patients are in separate places and communicate through OHCs (Wu and Lu, 2017). Various types of consultations, such as image-texting, telephone, video, and even live consultations, can effectively alleviate the difficulty and expense of medical treatment for patients. Online consultations also provide physicians with a new method of working without time and space restraints (Yin et al., 2022). In general, online patient consultations are an innovative approach to meeting escalating medical demand, enabling users to overcome boundaries of geography and time to provide more possibilities for choosing physicians around the world (Gong et al., 2020).

Recently, online healthcare consultation has received substantial attention from academics and healthcare practitioners (Chen et al., 2021). During COVID-19, Shah et al. (2021) utilized signaling theory to examine the influence of various online and offline signals and disease risk on patients’ choices of physicians for online consultations. Liu et al. (2019) investigated the influence of reviews on online consultation behaviors in OHCs based on 907 Chinese OHC website physicians. By contextualizing the valence framework, Yang et al. (2021) re-examined the effect of multidimensional trust on patients’ decisions to continue using an online medical consultation service. However, little research has examined patients’ online consultations from the perspective of the social support provided by physicians. Therefore, it is necessary to explore the important role of physicians’ social support in online healthcare consultation decision-making.

### Social support theory

Online healthcare communities are platforms with social networking features, while they allowing users to express health-related queries and experiences and offer social, emotional, and informational support (Eysenbach et al., 2004). Social support exchange is described as information that makes people feel cared for and valued, esteemed, and part of a network of social obligations (Cobb, 1976). Different people have different expectations for the interactions that take place in a social exchange depending on the circumstances. Consequently, OHCs are sites for the interchange of verbal and nonverbal cues providing emotional support, information, or recommendations to alleviate worry or tension (Walther and Boyd, 2002). Individuals’ information transmission could make social support more accessible, with the content affected by the type of support (Lin and Kishore, 2021). Social support includes multiple dimensions. Existing studies believe that social support in general contains informational, emotional, and companionship support (Wang et al., 2021), esteem support, tangible support, network support (Cutrona and Suhr, 1992), and experiential support (Lin and Kishore, 2021). However, online social support includes informational support and emotional support in the forms of kindness and sympathy (Chen et al., 2020; Mirzaei and Esmaeilzadeh, 2021). Some studies on OHCs focus on the impacts of informational and emotional support (Eysenbach et al., 2004). Lin and Kishore (2021) investigated social support in three dimensions: informational support, experiential support, and emotional support. This study aims to explore the impact of social support on patient consultation from physician’s perspectives, and since experiential support is from the patient’s perspective, it is excluded for this study.

The emergence of OHCs has profoundly affected patient consultations, allowing physicians and patients to communicate in new ways and access crucial information to meet each other’s social requirements (Eysenbach et al., 2004). In OHCs, physicians post articles on illness treatment and preventative approaches and present their medical or academic titles to bolster patients’ beliefs in the information. Patients can utilize this information to select a physician to treat their ailment (Ouyang et al., 2022). In addition, when ill patients crave the attention of others, and the physician’s demeanor or words might help them feel at ease. Therefore, informational and emotional support plays an important role in OHCs as important dimensions of social support. Previous studies explore the impact of physicians’ social support on disease treatment. For example, Thoits (1982) explored how social support helps patients cope with stressful circumstances (Thoits, 1982), and Cohen and Wills (1985) studied the connection between social support and psychological well-being (Cohen and Wills, 1985). Kiyohara et al. (2001) demonstrated that patients with serious conditions expected more social support during online physician-patient interactions or during physician assistance (Kiyohara et al., 2001). McCorkle et al. (2008) showed that social support boosts people’s well-being and relieves psychiatric symptoms (McCorkle et al., 2008). Later, researchers gradually began to take notice of the influence of social support on consumer behaviors in OHCs. Yang et al. (2015) correlation between social support and patient satisfaction in patients with varying levels of disease risk (Yang et al., 2015). Both Frow et al. (2016) and Saggi and Jain (2018) examined the function of social support in treating illness severity as a regulatory factor. Patients and physicians can contribute to the value-creation process through online consultation. However, there is still a lack of research on the impact of physicians’ social support during patient consultation. Therefore, social support theory is adopted in this study to examine the impact of physicians’ social support on patient consultations in OHCs.

## Research model and hypotheses

**Figure 1:**
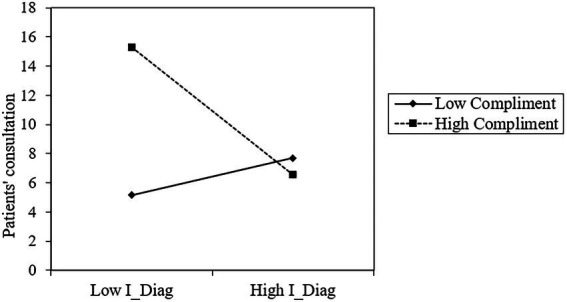
Moderating effect (Information diagnosticity).

As shown in Figure 1, we have created a research model of dimensions of physician’s social support and patient consultations to overcome the limits of prior studies. Social support has caught the attention of researchers due to its positive impact on purchase intent in a social commerce context (Makmor et al., 2018; Hu et al., 2019). Similarly, in OHCs, research has been conducted on social support and patients’ engagement (Wang et al., 2017), and the effect of linguistic signals on online social support (Chen et al., 2020). However, research on the relationship between relevant dimensions of social support and patient consultation is still limited. Thus, this study explores the relationship between social support and patient consultations. Based on the social support theory, we first discuss the three dimensions of physician social support: information diagnosticity, source credibility, and emotional support. Secondly, the study verifies the direct impact of these three dimensions on patient consultation. Thirdly, based on the social influence theory, the moderating effect of patients’ compliments on the dimensions of physician’s social support and patient consultations was verified.

### Informational support and patient consultation

Informational support is one of the dimensions of social support theory (Wang et al., 2021). Personal experience, recommendation, suggestion, and feedback are all behaviors associated with information support (Zhao et al., 2020). Individuals can benefit from the informational support of others, especially when the support is related to the stressful events they are experiencing (Ma et al., 2021). In OHCs, the act of providing and exchanging information related to medical technologies and medical procedures, such as illness (diagnosis, prescription, treatment, and records while being treated), hospitalization, enrolment, and others, is referred to as informational support (Wang et al., 2020). In other words, informational support refers to physicians answering concerns about illness prevention and treatment. Information and emotional support are the primary objectives of physician online consultation services (Wang et al., 2020). Patients have different perceptions of the various channels in OHCs. Some OHCs are intended to assist patients in dealing with emotions, while others aim to educate patients about their diseases (Mirzaei and Esmaeilzadeh, 2021). In OHCs, physicians tend to focus more on patients’ information needs than their emotional needs (Ma et al., 2021). In this study, informational support has two dimensions: information diagnosticity and source credibility.

Information diagnosticity or perceived diagnosticity (Wang and Chang, 2013; Kim and Youn, 2019), reflects the value of a piece of information and depends on how much the information aids in patient in decision-making (Andrews, 2013). The amount, intensity, and discernibility of the information contribute to its diagnostic usefulness (Andrews, 2013). The more information provided, the higher the effectiveness, and the more it increases patients’ confidence. Consumer choice decisions are made easier when information can be easily assessed for authenticity. For example, Lee et al. (2021) studied crowdfunding websites and found that positive information diagnosticity can act as an intermediary to influence consumers to actively participate in a crowdfunding project. Filieri (2015) shows that the amount of information partially impacts the information diagnosticity and ultimately affects consumers’ information adoption. Physicians can benefit from the ability of OHC to circumvent regional limitations and promptly reach patients. As a result, many physicians are turning to the Internet to disseminate their expertise (Zhang et al., 2022). Physicians can use OHCs to offer patients articles on the treatment and prevention of disease. Because patients lack specialized healthcare skills and have difficulty finding relevant medical information, it helpful to have access to the information online (Carlsson, 2000). More medical information may improve patients’ perceptions of the quality of their physicians, which may alter their consultation selections. Physicians can freely offer consultations and publish articles on illness prevention and treatment to attract more visits or consultations, which patients can access at any time and place (Zhang et al., 2022). Therefore, information diagnosticity of physicians in OHCs may promote patient consultation behaviors. The following hypotheses are proposed.

*H1*: Information diagnosticity positively affects patient consultations.

Source credibility, information credibility (Fan and Lederman, 2018; Ali et al., 2020), or message credibility (Xu et al., 2021) all refer to the trustworthiness of the information and recipients consider the source of information to be credible mainly because the information disseminator is an expert, despite the fact that the actual information may or may not be (Dholakia and Sternthal, 1977; Sussman and Siegal, 2003). As a general rule, people believe that expert opinions are correct and that experts are reliable authorities on the information to be used for making decisions (Bonner et al., 2006). Thus, source credibility will affect users’ attitudes and behaviors (Sussman and Siegal, 2003). Many previous studies on source credibility focus on how consumers judge the credibility of information sources in the online shopping environment. For example, in e-commerce studies, Zhang et al. (2014) investigated the impact of online reviews on intention to purchase by examining source credibility. In advertising studies, Zha et al. (2015) found that source credibility can assist the development of positive consumer attitudes about online products when used for online advertising. Dedeoglu (2019) showed that tourists’ opinions of the credibility of social media are linked to the value of non-participant shared content. Furthermore, in previous studies on OHCs, Robertson-Lang et al. (2011) found that trustworthiness was a key factor in determining whether the elderly searched for online health information. In OHCs, as the patients are non-professionals, they lack the knowledge related to medical treatment, so they can only judge the professional degree of the physician by some official endorsement, such as the title of the physician. Therefore, many high-quality OHC platforms currently require physicians to be certified under their real names (Liu et al., 2014; Zhang et al., 2020) and encourage physicians to attach their professional or academic titles to their profiles as to improve their credibility with patients. In addition, patients prefer to choose physicians with the best reputations, numerous professional and academic titles, and wealth of experience (Shah et al., 2021). Some studies of OHCs show that source credibility helps patients adopt information. Fan and Lederman (2018) showed that OHC patients will use health information sources that they trust and think are credible. Zhang et al. (2020) used online and offline physician experience, hospital location, and level of credibility to examine their impact on the knowledge adoption of Chinese OHC users. Therefore, the source credibility of physicians in OHCs may increase patient consultation behaviors. Hence, the following hypothesis is proposed.

*H2*: Source credibility positively affects patient consultations.

### Emotional support and patient consultation

Emotional support refers to intentional verbal behaviors used to reduce the emotional distress of others (Burleson, 1985). OHCs offer a way to seek and share knowledge and emotional support without being restricted by time, space, or geographical location (Mirzaei and Esmaeilzadeh, 2021). In OHCs, nothing is more important than emotional support for improving patients’ health (Ma et al., 2021). Patients want their physicians to demonstrate professional expertise while delivering personal care and compassion (Schattner et al., 2004). Many patients require emotional support (Vlahovic et al., 2014), such as understanding, encouragement, empathy, affection, affirmation, validation, care, and concern (Nakikj and Mamykina, 2017). In the context of OHCs, physicians express emotional support *via* homepage greeting messages. Patients can determine whether the physician can provide emotional support by reviewing their greeting messages. Some physicians, for example, write encouraging and heart-warming messages, while others write a few words about themselves or may not bother to write greeting messages at all. Chen et al. (2019) developed a model that integrates patients’ network status, informational support, emotional support, and downstream individual-level health knowledge with attitudes in OHCs. Atanasova et al. (2018) adopted the grounded theory approach to explore emotional support. Patients and physicians believe that the main benefit of online professional interaction is providing emotional support for users. Abedin et al. (2020) discovered that many patients also actively posted on OHC to provide other members with knowledge and emotional support. Since patients visit OHCs in search of emotional support, it has been shown that a pleasant emotion tone in physicians’ information or messages influences patients’ decisions positively (Yan and Tan, 2014; Ouyang et al., 2022). Wang et al. (2017) explored the positive influence of emotional support on OHCs participation through the crawler technology mobile network forum data. Therefore, the emotional support of physicians in OHCs may promote patient consultation behaviors, leading to the following hypothesis.

*H3*: Emotional support positively affects patient consultations.

### Moderating effect of patients’ compliments

Social influence theory discusses how one’s attitude or evaluative orientation can be affected and the theory is used to understand group interactions (Kelman, 1958). Social influence is related to the effects of peer thoughts and activities on other people’s behaviors (Alam et al., 2020). Kelman (1958) proposed that social influence theory has three social processes that influence people’s behavior: compliance (influencing others’ expectations), internalization (aligning one’s aims with the goals of others), and identification (self-perception in the context of the group’s characteristics). According to decades of human research, social influence from others is a major source of behavior change (Tunçgenç et al., 2021). Researchers have used social influence theory to study how social influence predicts willingness to participate in virtual communities and thus predicts actual behavior (Dholakia et al., 2004; Zhou, 2011). In OHCs, there is a large information imbalance between physicians and patients (Kromidha and Li, 2019), making it difficult for patients to choose a physician. Although information asymmetry is more serious than it is in face-to-face consultation (Laugesen et al., 2015), the social influence of other patients can help reduce the degree of patients’ understanding of the information given by physicians to reduce the degree of information asymmetry (Ho and Wei, 2016). When patients use a telemedicine service, their comments are visible to everyone online and can motivate other patients to adopt the technology. For example, telemedicine services can communicate the credibility of articles on disease prevention and treatment, the physician’s level, and whether the physician behaves gently and reliably and cares about their patients. Endorsement from other patients, such as *via* e-WOM, is also very important for evaluating these issues. Consequently, the social influence of other patients is essential to telemedicine services (Kamal et al., 2020). Most influential are other patients’ compliments.

Compliments refer to favorable assessments expressing something positive about another person (Wolfson and Manes, 1980). Compliments are powerful sources of feedback (Kraft and Martin, 2001). In the context of OHCs, compliments indicate acknowledgment of a physician’s services and hard work which enhances their reputation (Wu and Lu, 2017). After physicians provide services to patients in OHCs, physicians can get external compliments, such as electronic votes, letters of thanks letters, and e-gifts from patients (Liu et al., 2022). In the setting of OHCs, information is abundant, frequently leading patients seeking knowledge to have information overload (Swar et al., 2017). The limited-capacity theory holds that a person can only pay attention to a limited number of things at a time and that attention cannot be shared with other, lower-priority tasks (Xia et al., 2020). In addition, consumers devote fewer attentional resources when confronted with a large amount of information, which increases the difficulty of choosing (Peng et al., 2021). However, other patients usually need more effort, time, knowledge, and even money (gifts) to compliment one physician, which seems more credible for patients’ decision-making than physicians (Wu et al., 2020). Since compliments are a social influence, the relationship between physicians’ social support and patient consultations may be weakened by other patients’ compliments. Specifically, when other patients’ compliments are high, patients facing choices will be more likely to identify the social influence of these patients and make convergent choices, thus weakening the positive impact of physicians ‘social support on patient consultation. When other patients’ compliments are low, physicians ‘social support is particularly important for patients’ decision-making, thus strengthening the positive impact of physicians ‘social support on patient consultation. This leads to the following hypotheses.

*H4*: Patients’ compliments weaken the positive relationship between information diagnosticity and patient consultations.

*H5*: Patients’ compliments weaken the positive relationship between source credibility and patient consultations.

*H6*: Patients’ compliments weaken the positive relationship between emotional support and patient consultations.

## Methodology

### Data and measures

A Python spider was used to retrieve the data set from Haodf.com.^1^ Haodf.com, one of China’s largest OHCs, was founded in 2006 and has grown rapidly since then. A range of medical issues can be dealt with using the Haodf.com App, the P.C. website, the mobile website, and other platforms. As of October 202, more than 740,000 patients have been handled by the wide network of highly competent medical providers registered with Haodf.com, with 240,000 physicians registered and 73% of these physicians working in a large highly ranked hospital in China (Good doctor online, 2022). In this study, we consider 14 diseases, divided into two categories: high risk of death and low risk of death. High risk of death: (1) diabetes, (2) coronary heart disease, (3) hypertension, (4) Parkinson’s disease, (5) lung cancer, (6) liver cancer, and (7) breast cancer. Low risk of death: (1) infertility, (2) menstrual disorders, (3) prostatitis, (4) hepatitis B, (5) depression, (6) pharyngitis, and (7) pneumonia in children. After deleting the entries with “space” or “missing value,” we obtained data from 2,982 physicians. The data contains physicians’ personal and consultation profiles and patients’ feedback.

This study consists of 10 variables: one dependent variable, three independent variables, one moderating variable, and five control variables (see Table 1). On OHCs, the number of patient consultations is an accurate measurement of the online performance of physicians (Zhang et al., 2022). Thus, the dependent variable is the total number of patient consultations (*Consult*). The independent variables are Information Diagnosticity (*I_Diag*), Source Credibility (*S_Cre*), and Emotional support (*Emotion*). For the reasons mentioned in the hypotheses, we collected several health-related articles to measure information diagnosticity, physicians’ titles to measure source credibility, and the length of greeting messages to measure emotional support. The moderating variable is patients’ compliments (*Compliments*), representing the standardized average of digital gifts, votes, and thank-you letters (Wu et al., 2020). The control variable is physicians’ gender (*Gender*), male or female; the hospital type (*H_type*), public or private; the hospital level (*H_level*), which is AAA level; specialist hospital (*H_Special*), indicating a hospital for the treatment of the particular disease; and disease severity (*D_Severity*), divided by degree of mortality.

**Table 1:** Descriptive statistics.

Variables	Description	Mean	S.D.	Min	Max	
**Dependent Variable**						
Consult	The number of patients’ consultation in total	4581.30	5671.15	73	71,678	
**Independent Variable**						
I_Diag	Number of health-related articles	15.66	65.83	0	2,018	
S_Cre	The medical titles of the physician were stratified into 4 stages, 1 = the resident physician, 2 = the attending physician, 3 = associate chief director, 4 = chief director.	3.25	0.74	1	4	
Emotion	The length of greeting message	113.94	202.95	0	3,975	
**Control Variable**						
Gender	Dummy variable indicating physicians’ gender 0 = Male, 1 = Female	0.34	0.47	0	1	
H_type	Dummy variable indicating the hospital type 0 = Private, 1 = Public	0.99	0.08	0	1	
H_level	Hospital level: the scale of 1 to 3, with 1 being the lowest (1A or 1B) and 3 the highest (3A or 3B hospitals)	2.99	0.12	1	3	
D_severity	Dummy variable indicating the mortality of the disease 0 = low, 1 = high	0.37	0.48	0	1	
H_Special	Dummy variable indicating whether the hospital is a specialized hospital 0 = Specialized, 1 = General	0.67	0.47	0	1	

### Model specification

Main models use the OLS regression with fixed effect estimations. As the dependent variable is numeric data with non-negative integers (number of patient consultations a physician has received), we use negative binomial regression models to test the robustness. We use OLS regression models with the total number of patient visits as an alternative dependent variable to patient consultations to test the robustness because patients can choose consultations only after visiting. To test the model, this study analyzed the main equations below:


$$\begin{array}{l} \log{\left(Consult\right)}_{i}=\alpha_{0}+\beta_{1}\log{\left(I\_Diag\right)}_{i}\\ \;\;\;\;+\beta_{2}S\_Cre_{i}+\beta_{3}\log{\left(Emotion\right)}_{i}\\ \;\;\;\;+\beta_{4}D\_severity_{i}+\beta_{5}Gender_{i}\\ \;\;\;\;+\beta_{6}H\_type_{i}+\beta_{7}H\_level_{i}\\ \;\;\;\;+\beta_{8}H\_special_{i}+\mu_{i}+E_{i}\;\left(1\right) \end{array}$$

$$\begin{array}{l} \log{\left(Consult\right)}_{i}=\alpha_{0}+\beta_{1}\log{\left(I\_Diag\right)}_{i}\\ \;\;\;\;+\beta_{2}S\_Cre_{i}+\beta_{3}\log{\left(Emotion\right)}_{i}\\ \;\;\;\;+\beta_{4}Compliment_{i}\\ \;\;\;\;+\beta_{5}\log{\left(I\_Diag\right)}_{i}*Compliment_{i}\\ \;\;\;\;+\beta_{6}S\_Cre_{i}*Compliment_{i}\\ \;\;\;\;+\beta_{7}\log{\left(Emotion\right)}_{i}*Compliment_{i}\\ \;\;\;\;+\beta_{8}D\_severity_{i}+\beta_{9}Gender_{i}\\ \;\;\;\;+\beta_{10}H\_type_{i}+\beta_{11}H\_level_{i}\\ \;\;\;\;+\beta_{12}H\_special_{i}+\mu_{i}+E_{i}\;\left(2\right) \end{array}$$

For each of the effects, there is one constant term, individual effects $,\left(\mu_{i}\right),$ and one residual error term ( $E_{i}$ ). We perform log transformations on the *Consult*, *I_Diag,* and *Emotion* datasets, all of which have skewed distributions (skewness = 3.211, 18.458, and 8.382, respectively). In equation 1, three dimensions of social support, information diagnosticity (*I_Diag*), source credibility (*S_Cre*), and emotional support (*Emotion*), are used to determine whether they have positive effects on patient consultations. In equation 2, compliments (*Compliments*) from other patients may have moderating effects on the relationship between dimensions of social support and patient consultations. Furthermore, robustness checks employ alternative negative binomial and OLS regression models with the total number of patients’ visits as an alternative dependent variable. We use a log transformation on the total number of patient visits with a skewed distribution (skewness = 5.398) in OLS regression robustness models.

### Results

When it is less than or equal to 0.190, the coefficient of determination (*R*-squared) value is regarded as weak (Newsted et al., 1998). In this study, the coefficient of determination (R-squared) of model 2 = 0.282 and model 3 = 0.557, indicating that model 3 has good prediction accuracy.

Table 2 shows the correlation coefficients among all variables. The results show that *I_Diag* (*r* = 0.418, *p* < 0.001), *S_Cre* (*r* = 0.272, *p* < 0.001), and *Emotion* (*r* = 0.323, *p* < 0.001) are significantly positively correlated with the dependent variable *Consult*. In addition, the variance inflation factor (VIF) all the models’ is tested to estimate multicollinearity. The results show that the average VIF value is 1.12, which is lower than 10, indicating that multicollinearity is not a serious problem (Mason and Perreault, 1991).

**Table 2:** Correlation coefficient matrix.

	1	2	3	4	5	6	7	8	9
log(Consult)	1.000								
log(I_Diag)	0.418^***^	1.000							
	(0.000)								
S_Cre	0.272^***^	0.151^***^	1.000						
	(0.000)	(0.000)							
log(Emotion)	0.323^***^	0.401^***^	0.124^***^	1.000					
	(0.000)	(0.000)	(0.000)						
D_severity	−0.154^***^	0.030^*^	0.081^***^	0.019^*^	1.000				
	(0.000)	(0.105)	(0.000)	(0.290)					
Gender	−0.078^***^	−0.187^***^	0.054^**^	−0.124^***^	−0.160^***^	1.000			
	(0.000)	(0.000)	(0.003)	(0.000)	(0.000)				
H_type	−0.006	−0.039^*^	0.002	−0.016^*^	0.039^*^	−0.033^*^	1.000		
	(0.752)	(0.033)	(0.926)	(0.389)	(0.033)	(0.070)			
H_level	0.014^*^	−0.014^*^	0.001	−0.002	0.049^**^	−0.061^***^	0.385^***^	1.000	
	(0.441)	(0.443)	(0.941)	(0.930)	(0.008)	(0.001)	(0.000)		
H_Special	−0.027^*^	0.031^*^	0.024^*^	0.018^*^	0.095^***^	−0.063^***^	0.061^***^	0.029^*^	1.000
	(0.143)	(0.094)	(0.184)	(0.331)	(0.000)	(0.001)	(0.001)	(0.115)	

*p* values in parentheses.

* *p* < 0.05;

** *p* < 0.01;

*** *p* < 0.001.

As shown in Table 3, models 1–3 map is the main models of OLS regressions. In model 1, the control variables were introduced. The result suggested that most control variables have good significance. In model 2, the control variables and dependent variables were introduced. Firstly, the result suggests that *I_Diag* (*β* = 0.276, *p* < 0.001) has a significant positive impact on *Consult.* Hypothesis 1 is supported, indicating that information diagnosticity positively affects patient consultations. Secondly, the result suggests that *S_Cre* (*β* = 0.335, *p* < 0.001) has a significant positive impact on *Consult.* Hypothesis 2 is supported, which indicates that source credibility positively affects patient consultations. Thirdly, the result suggests that *emotion* (*β* = 0.092, *p* < 0.001) has a significant positive impact on *Consult.* Hypothesis 3 is supported, indicating that emotional support positively affects patient consultations.

**Table 3:** Regression result.

	Main models			Robustness models			
	Model 1	Model 2	Model 3	Model 4	Model 5	Model 6	Model 7
Constant	7.632^***^	5.474^***^	7.113^***^	6.085^***^	8.143^***^	8.299^***^	10.197^***^
	(0.39)	(0.38)	(0.38)	(0.53)	(0.55)	(0.61)	(0.60)
D_severity	−0.402^***^	−0.447^***^	−0.414^***^	−0.399^***^	−0.422^***^	−0.543^***^	−0.505^***^
	(0.04)	(0.04)	(0.03)	(0.04)	(0.03)	(0.05)	(0.04)
Gender	−0.248^***^	−0.096^*^	0.050^*^	−0.118^**^	0.031^*^	−0.174^**^	−0.006
	(0.04)	(0.04)	(0.03)	(0.05)	(0.04)	(0.05)	(0.05)
H_type	−0.129	0.093	−0.082	0.166^*^	−0.083	0.395^*^	0.190
	(0.29)	(0.23)	(0.20)	(0.21)	(0.19)	(0.42)	(0.39)
H_level	0.168^*^	0.206^*^	0.059	0.208^*^	−0.084	0.370^*^	0.208^*^
	(0.16)	(0.15)	(0.14)	(0.19)	(0.19)	(0.23)	(0.22)
H_Special	−0.036^*^	−0.071^*^	−0.071^*^	−0.088^*^	−0.057^*^	−0.054^*^	−0.057^*^
	(0.04)	(0.04)	(0.03)	(0.05)	(0.03)	(0.05)	(0.05)
log(I_Diag)		0.276^***^	0.160^***^	0.247^***^	0.155^***^	0.491^***^	0.362^***^
		(0.02)	(0.01)	(0.02)	(0.01)	(0.02)	(0.02)
S_Cre		0.335^***^	0.207^***^	0.284^***^	0.157^***^	0.697^***^	0.546^***^
		(0.02)	(0.02)	(0.03)	(0.02)	(0.04)	(0.03)
log(Emotion)		0.092^***^	0.051^***^	0.082^***^	0.038^***^	0.151^***^	0.103^***^
		(0.01)	(0.01)	(0.01)	(0.01)	(0.01)	(0.01)
Compliments			1.557^***^		1.599^***^		1.996^***^
			(0.15)		(0.15)		(0.20)
log(I_Diag)^*^Compliments			−0.134^***^		−0.144^***^		−0.173^***^
			(0.02)		(0.02)		(0.03)
S_Cre^*^Compliments			−0.093^*^		−0.092^*^		−0.149^**^
			(0.04)		(0.04)		(0.05)
log(Emotion)^*^Compliments			−0.046^**^		−0.041^**^		−0.061^**^
			(0.02)		(0.01)		(0.02)
City dummies	Yes	Yes	Yes	Yes	Yes	Yes	Yes
R2	0.036	0.282	0.557			0.397	0.555
adj. R2	0.033	0.279	0.554			0.395	0.553
N	2,982	2,982	2,982	2,982	2,982	2,982	2,982

Standard errors in parentheses.

* *p* < 0.05;

** *p* < 0.01;

*** *p* < 0.001.

**Figure 2:**
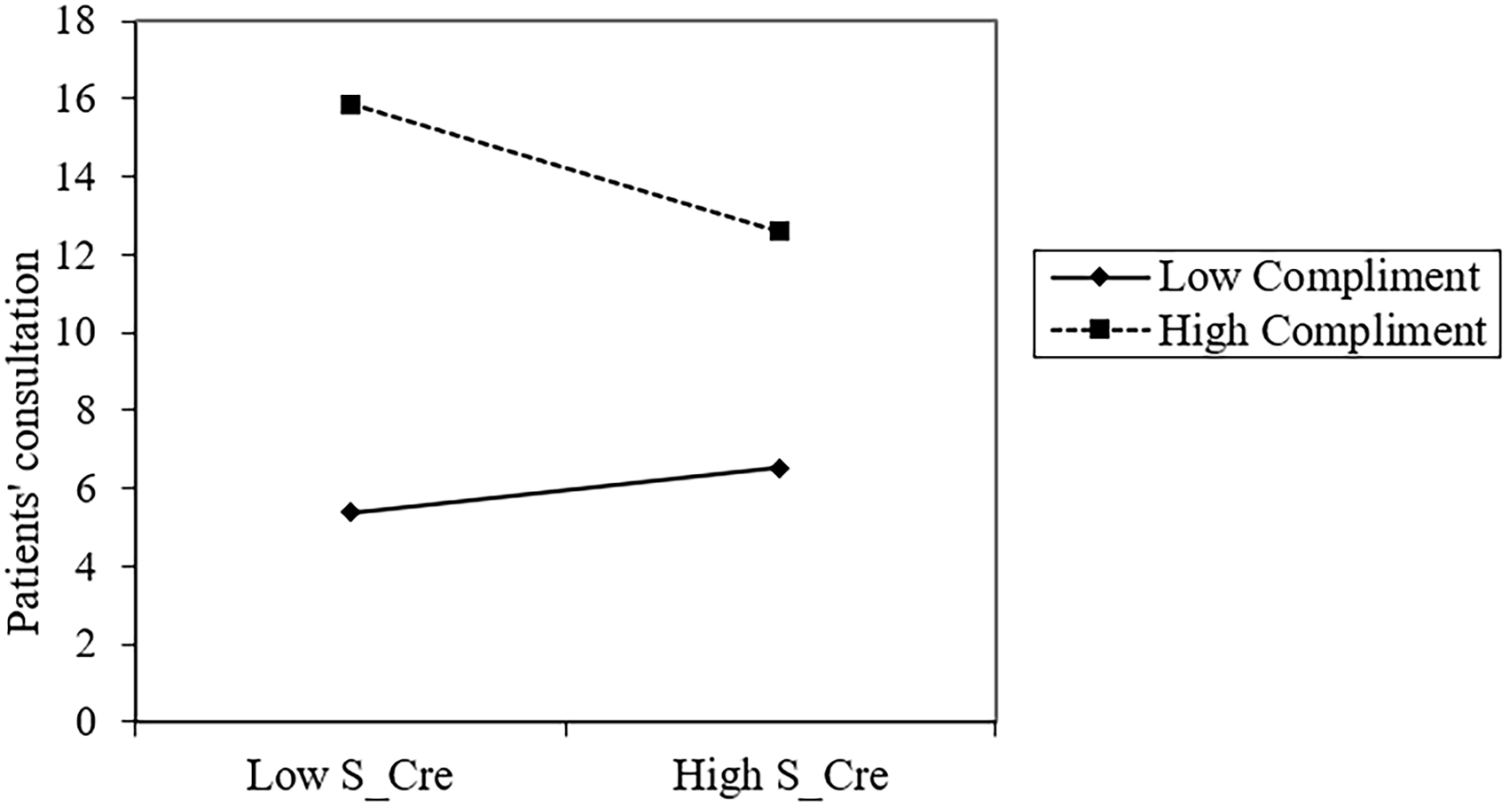
Moderating effect (Source credibility).

In model 3, the control, dependent, and moderating variables were introduced. Firstly, the result suggests that the interaction of *I_Diag* and *Compliments* (*β* = −0.134, *p* < 0.001) negatively affects *Consult.* As shown in Figure 2, I_Diag has a stronger impact on *Consult* when *Compliments* are low but a smaller impact when *Compliments* are high. Hypothesis 4 is supported, indicating that patient compliments weaken the positive relationship between information diagnosticity and patient consultations.

**Figure 3:**
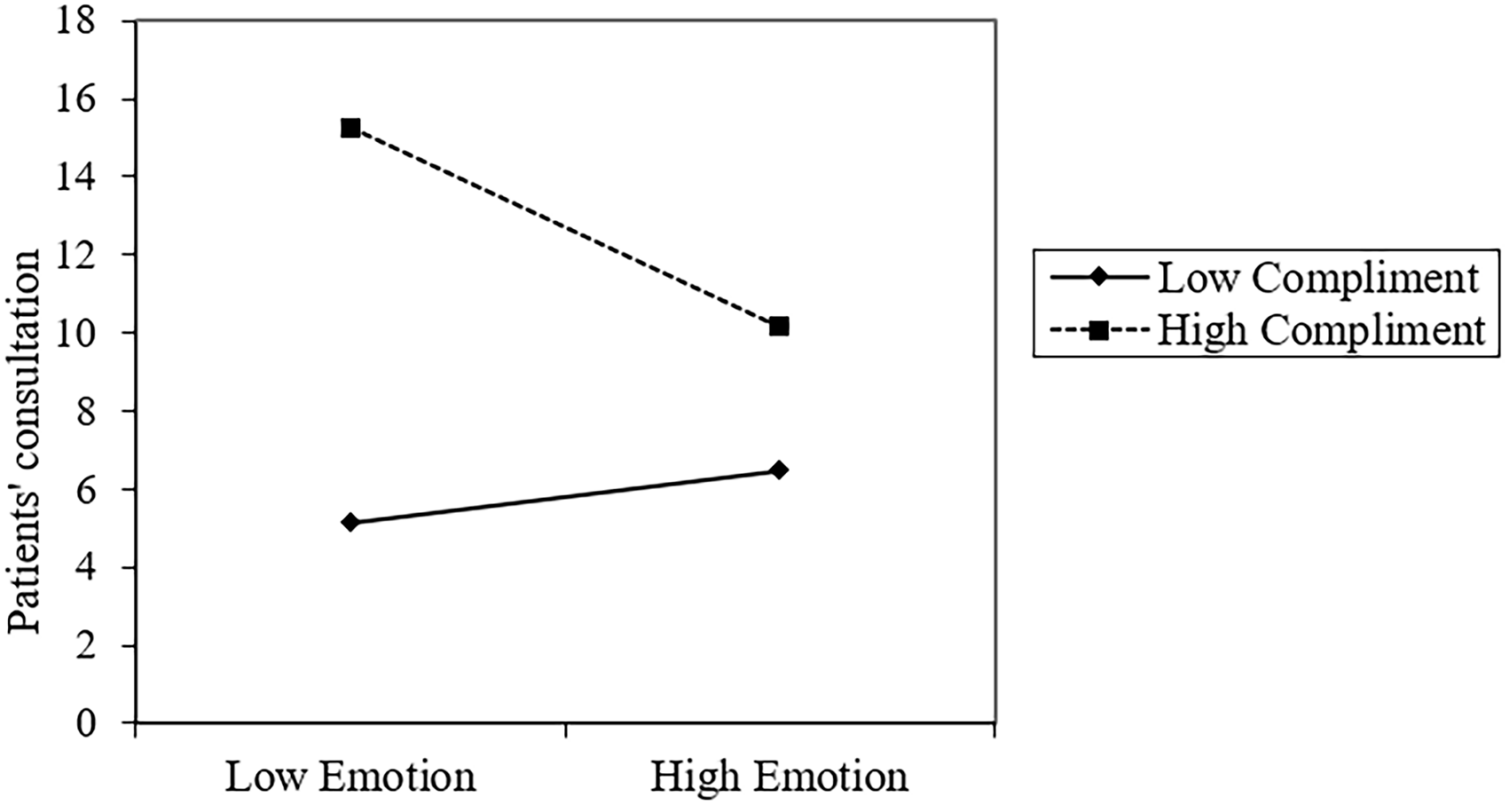
Moderating effect (Emotion support).

Secondly, the result suggests that the interaction of *S_Cre* and *Compliments* (*β* = −0.093, *p* < 0.05) significantly negatively affects *Consult.* As shown in Figure 3, S_Cre has a greater effect on *Consult* when *Compliments* is low but a smaller effect when *Compliments* is high. Patient compliments reduce the positive relationship between source credibility and patient consultation, supporting Hypothesis 5.

**Figure 4:**
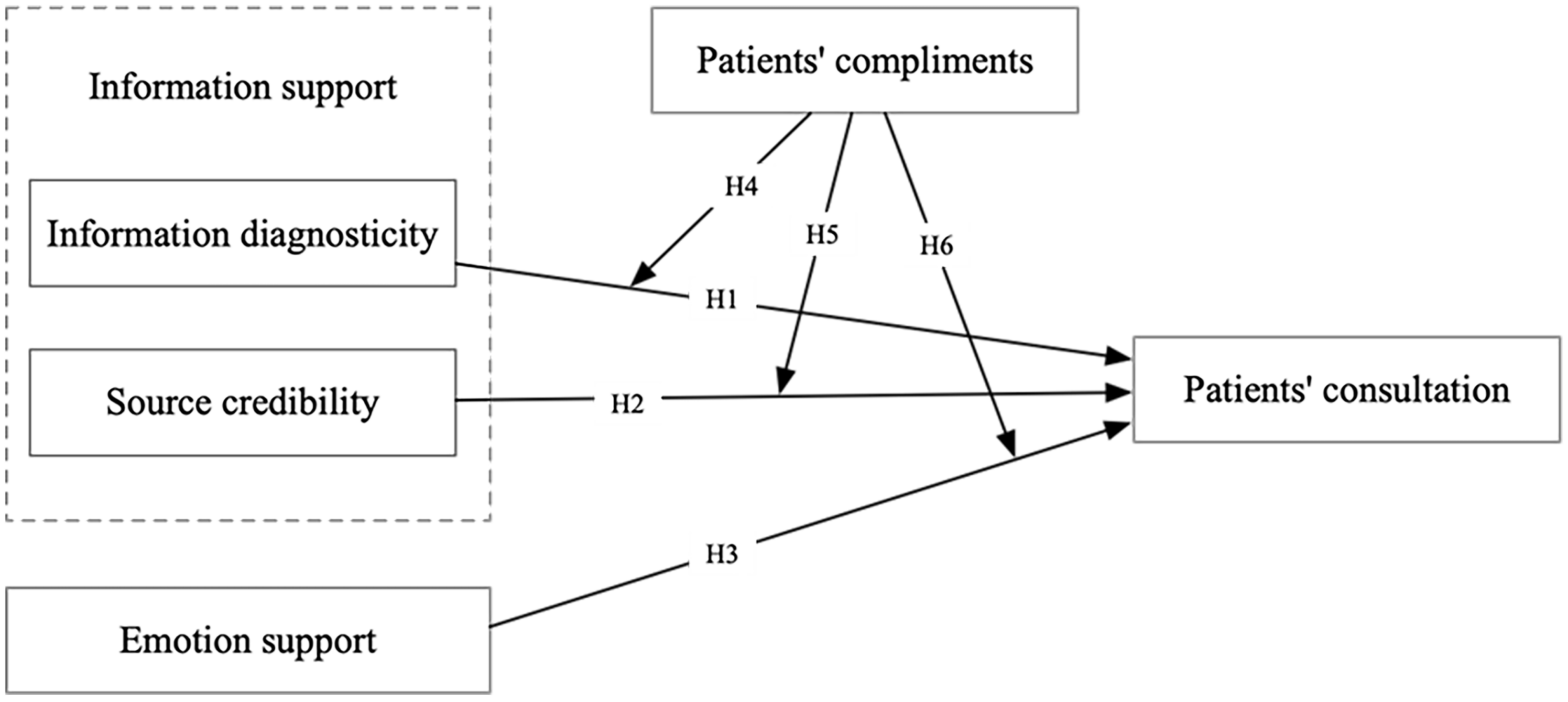
Concept model.

Thirdly, the result suggests that the interaction of *Emotion* and *Compliments* (*β* = −0.046, *p* < 0.001) negatively affects *Consult.* As shown in Figure 4, when *Compliments* is low, the effect of *Emotion* on *Consult* increases, while it diminishes when *Compliments* is high. Patient compliments reduce the positive relationship between emotional support and patient consultation, supporting Hypothesis 6.

### Robustness check

Models 4–7 show the robustness check Models used to check for the problems of autocorrelation and heteroskedasticity. In models 4 and 5, we use negative binomial regression as an alternative estimation method for the robustness check. The result of Model 4 shows that the *I_Diag* (*β* = 0.247, *p* < 0.001)*, S_Cre* (*β* = 0.284, *p* < 0.001), and *Emotion* (*β* = 0.082, *p* < 0.001) all have positive significant effects on *Consult.* The result of Model 5 shows that the interactions of *I_Diag* and *Compliments* (*β* = −0.144, *p* < 0.001), *S_Cre* and *Compliments* (*β* = −0.092, *p* < 0.05), and *Emotion* and *Compliments* (*β* = −0.041, *p* < 0.001) all have negative significant effects on *Consult.* In Models 6 and 7, we use the total number of patient visits as an alternative dependent variable method as the robustness check. The result of model 6 shows that the *I_Diag* (*β* = 0.491, *p* < 0.001), *S_Cre* (*β* = 0.697, *p* < 0.001), and *Emotion* (*β* = 0.151, *p* < 0.001) all have positive significant effects on *Consult.* The result of model 7 shows that the interactions of *I_Diag* and *Compliments* (*β* = −0.173, *p* < 0.001), *S_Cre* and *Compliments* (*β* = −0.149, *p* < 0.001), and *Emotion* and *Compliments* (*β* = −0.061, *p* < 0.001) all have negative significant effects on *Consult.* Consequently, the coefficients of the robustness check model and the main models are consistent.

## Discussion

In the context of OHCs, patients are concerned with choosing a competent physician. Physicians’ informational and emotional support plays an important role in these choices (Uchino, 2009). Based on social support theory and social influence theory, this study investigated the impact of information diagnosticity, source credibility, and emotional support on patient consultations given the interference of patients’ compliments. The results show that all hypotheses are supported. The study revealed four important key findings.

First, this study found that social support theory applies to patient consultation in the OHC environment. Diagnostic information such as disease prevention and treatment articles as a kind of informational support provided by physicians is positively correlated with patients’ decision-making. This means that physicians can attract more patients to purchase their services by improving their diagnostic information, for example, by writing good articles on disease prevention and treatment to meet the information needs of patients. Information diagnosticity positively affects patient consultation. This research result is consistent with the conclusion of Gurney et al. (2019), which shows that the information diagnosticity positively affected purchase intention in an online sales context.

Secondly, this study confirms the positive impact of source credibility on patient consultation. The reliability of physicians’ professional titles as sources of credibility can indicate the level of technical expertise of physicians. If the physician’s rank is high, patients are more likely to choose them and trust them because the rank represents the physician’s expertise. Due to the importance of having a reputable professional title and given that the OHCs platform also encourages physicians to be certified by their real name and attach their professional title, the results of the study also suggest that physicians provide their professional title on the OHCs profile whenever possible, to provide a high level of source credibility. This indicates that information source credibility positively affects patients’ choices for online consultations. Furthermore, the influence of information source credibility on patient consultation is consistent with that of the result of Farhadpoor and Dezfuli (2021) and Qi and Kuik (2022) in the context of electronic shopping.

Thirdly, this study confirms the positive impact of emotional support on patient consultation. The word count of the greeting message may be a reference value to measure the emotional support of physicians. If the physician’s greeting message had more words, patients were more likely to consult them because patients perceive that this represents the physician’s patience, gentleness, and care for the patient. Considering that OHC platforms all have this function, the research results suggest that physicians should write longer greeting messages to indicate more emotional support and attract more patients to care for patients. This result is consistent with the conclusion of Wang et al. (2019), which shows that emotional support is positively correlated with purchase intention in WeChat health product consumption.

Finally, we discuss the moderating effects of other patients’ compliments on the relationship between three dimensions of social support and patient online consultations. The results indicate that the influence of information diagnostics, source credibility, and emotional support on patients’ online consultations increases when other patients’ praises are low and diminishes when they are high. The possible reason is that other patients’ compliments and physicians’ social support have a substitution effect on patient consultations. When other patients’ compliments are high, these other patients may post more credible information than the physicians’ information. Therefore, the positive effect of physicians’ social support on patient consultations is weakened. When other patients’ compliments are low, patients can only rely on physicians’ online information to make decisions. So, physicians’ social support has a significant positive impact on patient consultations. Our research shows that physicians should try to get more positive feedback and compliments, which attract more patients. This result is consistent with Huang et al. (2003), indicating that customer compliments, such as word of mouth, significantly affect other customers’ decisions. Therefore, other patients’ compliments play an indispensable role in OHCs.

## Theoretical contribution

This study provides several theoretical improvements to the existing body of knowledge. First, this study contributes to the literature on social support by proposing the social support theory to perform the patient consultation process. Prior researchers have studied the merits of social support in OHCs, including its positive influence on psychological well-being (Kim et al., 2012) and improved health outcomes (Eaker, 2005). To our knowledge, the theory has not been used to analyze patients’ consultation behavior in OHCs. Thus, this study contributes to the literature on social support theory by bringing the theory to explore the consultation mechanism of patients.

Second, this study improves the discussion of social support dimensions in OHCs. Previous studies rarely explored the social support dimension in OHCs. Our findings indicate that physicians’ informational support (information diagnostic and source credibility) and emotional support are positively associated with patient consultations, providing a novel angle for investigating how physicians’ social support affects patient consultations on OHC platforms.

Finally, this study contributes to the literature on patients’ compliments by revealing the moderating influence of patients’ compliments on the relationship between three dimensions of social support and patient consultations in OHCs. Our study demonstrates that patients’ compliments moderate the effect of social support (informational and emotional support) on patient consultations. When presented with vast information, consumers would dedicate fewer attentional resources, increasing choice difficulty (Peng et al., 2021). At this time, other patients’ compliments play an essential role since it is easy for patients to trust it in the physician selection process (Wu et al., 2020). Consequently, our findings contribute to distinguishing the potential signal effects of other patients or physicians.

## Practical contributions

This study has several implications for OHC practitioners and platform administrators. First, our findings indicate that physicians’ social support enhances patient consultation. Patients can be drawn by physicians’ social support, both informational and emotional support. For instance, patients evaluate the physicians’ information on the homepage of the OHC platform when deciding whether to contact the physician. Therefore, physicians should focus on their social support abilities to attract more patients for consultations.

Second, this study reveals that patients’ compliments significantly affect the consultation behavior of other patients. Patients pay attention to the number of digital gifts, votes, and thank-you notes posted by previous patients. These factors influence how patients choose a physician to consult. For example, physicians with a low level of patient compliments should pay greater attention to their social support capacity. Hence, as key participants in OHCs, physicians should be aware of previous patients’ compliments and seek more positive feedback.

Finally, platform administrators might employ a variety of ways to assist patients in identifying distinct physician groups. Platform administrators can implement tactics to encourage more patients to visit the homepages of physicians with high social support or positive patient feedback. In addition, platform administrators should highlight the possible benefits, such as social and economic rewards, of social support to motivate physicians to write warm greeting messages or publishing more health-related articles.

## Limitations and future research

This work yielded intriguing discoveries and made theoretical and practical contributions, yet it has multiple drawbacks. First, the study’s conclusions are based on data from China, which may restrict their applicability to other nations. To test the validity of our findings, future studies should incorporate data from several nations. Second, only cross-sectional data of the current period was captured by crawler technology in this study. Future studies may collect long-term panel data for more accuracy, such as 6 months of physicians’ home pages. Finally, this investigation is done on a single OHC website. Multiple website analyses are required to generalize our findings in the future.

## Conclusion

The COVID-19 pandemic poses significant challenges for the traditional healthcare system and affects how patients consult with physicians. The social support theory was used in this study to explore patient consultations and the moderating impact of patient compliments. To examine the relationship between physicians’ social support and patient consultation, this study collected cross-sectional data from a Chinese OHC platform named Haodf. This study found that patients’ compliments undermine the positive relationship between physicians’ social support and patient consultation. This study has practical implications for OHC users, both physicians and patients, and makes theoretical contributions to the literature on social support and patient consultations.

## Data availability statement

The original contributions presented in the study are included in the article/supplementary material, further inquiries can be directed to the corresponding author.

## Author contributions

ZX contributed to the current research ideas and performed the statistical analysis and contributed to improving the manuscript. XL and XY wrote the first draft of the manuscript and edited the revised manuscript and contributed to avoiding language errors. TO contributed to advising on the details. All authors contributed to the article and approved the submitted version.

## Conflict of interest

The authors declare that the research was conducted in the absence of any commercial or financial relationships that could be construed as a potential conflict of interest.

## Publisher’s note

All claims expressed in this article are solely those of the authors and do not necessarily represent those of their affiliated organizations, or those of the publisher, the editors and the reviewers. Any product that may be evaluated in this article, or claim that may be made by its manufacturer, is not guaranteed or endorsed by the publisher.

## Footnotes

^1^ www.haodf.com
